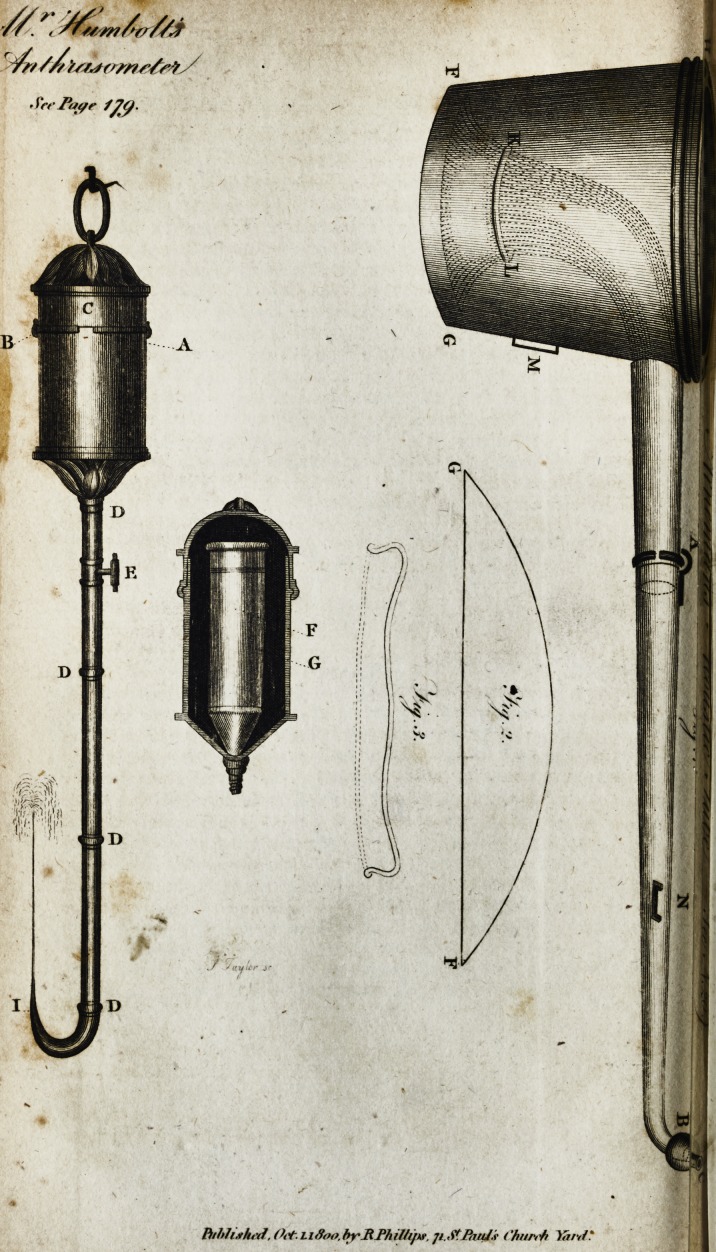# Book Reviews

**Published:** 1800-08

**Authors:** 


### CRITICAL RETROSPECT
### OF
### MEDICAL AND PHYSICAL LITERATURE.
### [ FOREIGN AND DOMESTIC. ]

*Tranfactions of a Society for the Improvement of Medical and Chirurgical Knowledge.*

[Continued from our laft Number, pp. 67—71.]

WE renew our Analyfis of this Volume with great pleafure, as it contains feveral important papers; and we felect the following, becaufe it furnifhes a very ufeful hint, and from which much advantage may be derived in the practice of midwifery.

" *Obfervations on the Management of Cafes in which the Face of the Child prefents towards the Os Pubis, by* JOHN CLARKE, M.D.

" Every perfon who has been engaged in the practice of midwifery knows, that if in labour the face of the child lies towards the fymphyfis pubis, confiderable difficulty is thereby frequently occafioned.

" More force muft be exerted by the woman, in order to expel the head fo fituated, and the labour will be protracted to a far greater length, than when the head is in the ufual pofition, with the occiput towards the os pubis.

" The ground of this difficulty is, becaufe in this fituation, the whole of the face muft defcend through the pelvis, before any part of the head can emerge from under the fymphyfis pubis, and becaufe the bones of the face are incapable of undergoing any alteration from preffure.

" On the other hand, when the occiput lies towards the os pubis, the different bones, of which the pofterior part of the head is compofed, are capable, in confequence of the incomplete offification at the futures, of very great alteration in their relative fituation, fo that the form of the whole head may become materially changed, and be better adapted to pafs out of the pelvis.

" Thus the fame head, from this variety in its pofition, may offer a very different degree of refiftance to the powers of expulfion.

" If the head fhould, unfortunately, be placed with the forehead towards the os pubis, and the woman fhould be ftrong, her exertions and pains muft be more violent in proportion to the increafed refiftance: but the labour, though prolonged very much beyond the ordinary bounds, may at length be finifhed by her own efforts. But in other inftances the difficulty may be fo confiderable, that her ftrength ftrength may be worn out without accomplifhing, the birth of the child; and fhe muft either remain undelivered, or artificial force muft be fubftituted for the natural powers, which are found to be defective.

"This unfavourable pofition of the head may be detected by a very little attention to the fituation of the anterior fontanelle, and of the futures.

"If, on examination, the anterior fontanelle be felt, and the fagittal future be found running from it towards one of the facro-illiac joints, or directly towards the concavity of the os facrum, there remains no doubt that the face will be born towards the fymphyfis pubis.

All the beft writers upon the practice of midwifery have taken notice of this caufe of difficulty in labour; but they have been contented with defcribing it, without fuggefting any means more efpecially fuited to this cafe. A reliance upon time, when the woman has ftrength, and the application of inftruments, when fhe has not, comprehend all the practical advice which is contained in their works.

"Chance firft led me to the knowledge of the fact, that, in fome cafes, this pofition of the head can be remedied without fubjecting the mother to any additional pain, or the child to any kind of danger.

"In a cafe where I had reafon to expect fome danger, I was defirous of knowing the precife pofition of the child's head, and whether it was in a fituation which would admit of delivering fafely with the forceps, if this fhould become indifpenfably neceffary. I found the face turned towards the groin, and on endeavouring to afcertain whether the ear could be felt, I was obliged to make a firm preffure againft the fide of the head with my finger.—In doing this it appeared to be moved a little. Aware of the great advantage which might arife to the patient if I could fucceed in bringing the occiput to the pubis, whether fhe were ultimately delivered by nature or by art, I continued to make preffure upon the fide of the head, till in the fpace of a few minutes the occiput was brought to the groin from the facro-iliac joint of the fame fide; the confequence of which was, that, inftead of the face, the occiput was born towards the pubis, and thus confiderable pain and difficulty were avoided.

"Reflecting upon the event of this cafe, I thought that the ready alteration in the pofition of the head might, in this inftance, depend upon the pelvis of the woman being very large, relatively to the volume of the child's head, and that a fimilar change could not be produced by the fame means in other cafes of a fimilar prefentation.—I determined, however, to make a trial in the next cafe which fhould occur. Another cafe foon occurring, this practice was attended with equal fuccefs.

"I have now met with fourteen cafes, in thirteen of which the practice has fucceeded; and as fome years have now elapfed fince the firft cafe, I think myfelf fully authorifed in recommending this method method to be always pursued, when the face is found in the situation above described. A great deal of pain, and much time, will be spared to the patient by these means.

"The manner of effecting the change is, by introducing one or two fingers between the side of the head, near the coronal suture, and the symphysis pubis, and pressing steadily against the parietal, or frontal bone, during a labour pain. When this is done, it will be found, in most cases, that the head yields to the pressure, till at length the occiput is brought to the groin. This being effected, the rest should be left to the natural efforts of the woman.

"It is unnecessary to observe, that this alteration will be more easily produced, when the face lies towards the groin, than when the sagittal suture runs directly backwards to the sacrum: but even in this case, the change of position may be effected with much more facility than I before hand supposed to be possible.

"When the pelvis happens to be large, or the head of the child small, as there will be more room for the head to turn, the difficulty of doing it will become proportionably less.

"In some instances, where the pelvis is small, or the head large, or where the Face is directly turned to the pubis, it may be impossible to change the direction: but these cases are comparatively rare; and as no harm can arise from it, the attempt may always be made."

"*A Case of Pregnancy, in which the Ovum had become diseased, and was entirely filled with small Hydatids. By* EVERARD HOME, *Esq.* F. R. S.

"THERE are many cases mentioned of the placenta losing its natural structure, and becoming a congeries of Hydatids; several of these have been published: but in all the instances on record, as far as I am acquainted with them, the patient had miscarried, and the placenta was examined after being separated from the uterus, so that the disease could not be exactly ascertained.

"The following case, in consequence of its melancholy termination, afforded an opportunity of the parts being examined in their natural situation, and of determining precisely the seat of the disease.

"Elizabeth Yeoman, thirty years of age, was delivered of her ninth child on the 22d of December, 1797, and recovered from her lying-in in three weeks, as she had usually done after former labours. She continued in perfect health till the 1st of August, 1798, when a flooding came on, which was supposed to be an uncommon flow of menstrual discharge; this continued for ten days; it created an alarm, and an accoucheur was consulted.

"He found, upon enquiry, that she had not menstruated before for twelve weeks, and there was an evident enlargement of the abdomen; he had therefore no doubt of her being pregnant, and thought she had advanced to the third month. From this explanation of the cafe, the prefent hæmorrhage appeared to be a flooding preceding abortion.

" The ordinary means for reftraining the flooding were ufed, and on the 14th it had entirely ftopped. She was now attacked by a vomiting, attended by a hot fkin, and a pulfe at 109. Thefe fymptoms were relieved by a gentle emetic, and faline draughts with rhubarb.

" On the 20th, there was a return of the vomiting, with fpafms over the whole abdomen, and the general bulk of the belly became much increafed; thefe fymptoms refifted all means ufed for their relief, and fhe died on the 22d, in the evening.

" Upon infpecting the body after death, the womb appeared to be of the fize that it ufually is five months after conception. The os tincæ was a little dilated, receiving with tolerable eafe the end of the finger.

" A longitudinal incifion was made through the anterior part of the uterus, extending from the os tincæ to the fundus; this incifion was continued through the membranes of the ovum, but no cavity was found, no fœtus prefented itfelf, and every part was occupied by an infinite number of hydatids of different fizes, from that of a pin's head to the fize of a common grape, fcarcely any of them being fo large as a full fized grape. They were attached to the neareft furface, and to every part of the amnion equally.

" Upon feparating the membranes compofing the ovum from the uterus, which was eafily done, the fituation of the placenta was readily afcertained; it was near the os tincæ on the pofterior furface of the uterus, and was unattached, a feparation having taken place during the patient's life time; this had occafioned the flooding, which gave the firft alarm refpecting her fafety. The furface next the uterus had not its ufual fhaggy appearance, but was nearly fmooth; it fcarcely projected beyond the membranes of the ovum, and was about two inches in diameter.

" The mafs of hydatids which filled the ovum were all connected with one another; were very tender, and readily broke down when handled. This mafs of hydatids was very different from thofe met with in the liver, which are fpherical, and thofe with long necks and heads, found in the brain of fheep; they were connected to the furface of the membrane, and to thofe hydatids with which they were united, by fhort pedicles or necks. To afcertain whether there was any appearance of a fœtus, I examined the whole with minute attention, but could not perceive the fmalleft remains either of one, or of a funis; nor could I, on the inner furface, diftinguifh any thing like the ordinary ftructure of the placenta.

" The hydatid ftructure of the placenta, as a difeafe to which that part is liable, is, I believe, very well known, and fpecimens of it are preferved in different collections of anatomical preparations. My attention in this cafe was directed to the inveftigation of the difeafe; and, from the facts I have ftated, it does not appear to be a change in the ftructure of the placenta, but a general affection tion of the amnion. When this disease takes place, the natural healthy actions for the support of the fœtus are so much impeded, that its growth is arrested. This evidently happened in a case published with an elegant engraving of the placenta and fœtus, by Dr. Denman; and when the patient does not early miscarry, the fœtus disappears; and in all the instances where miscarriage has taken place in a more advanced stage of the disease, I believe no fœtus had been found."

The following is an excellent example of the effect of the application of Gastric Juice to sores.

" *On the Use of the Application of Gastric Juice to Sores.* *By* JOHN HARNESS, M. D. *Physician to the Fleet.*

" MR. Thomas Corben, boatswain of his Majesty's ship Egmont, was, on the 31st of July, 1796, received into the Dolphin hospital ship, in consequence of a very bad ulcer of the right leg, which was fully six inches in length and four in breadth. The surrounding integuments were detached for some considerable way, and their margin, with very much the greater portion of the surface of the ulcer, was in a sphacelated state. The matter discharged was so extremely acrid, as to excoriate every part which it touched, and had insinuated itself through the whole length of the interstices of the gastrocnemius and soleus muscles. From between these muscles, which were detached from the bone nearly the whole length of the ulcer, a very large quantity of a most offensive matter was discharged, a considerable portion of which lodged in a cyst formed by the detached integuments on the posterior and inferior parts of the leg.

" There was a very great tension the whole length of the extremity.—The general health was much impaired.

" Having, in the course of a long and extensive practice, been too frequently a witness to the inefficacy of the applications hitherto made use of in the navy in the treatment of scorbutic ulcers (viz. cytric acid, bark, myrrh, rhubarb, and opium, separately or variously combined) and more particularly in warm climates, where sphacelus and erosions have taken place in any considerable degree, to which every seaman is peculiarly liable; I was induced, from the very unfavourable appearance of the case above related, to have recourse, with the concurrence of Mr. Gray, surgeon of the hospital ship, to the *Gastric Fluid of graminivorus animals*, which I knew could be readily obtained from the bullocks and sheep killed daily for the use of the fleet. From a bullock killed in the evening, nearly three pints of the juice were obtained, with part of which the surface of the ulcer was washed, as well as all the sinuses, by injecting the fluid into them in different directions by a syringe, with a long and slender tube. Superficial dressings of lint were then applied, and particular attention was paid to prevent, by compresses and bandages, a further insinuation of matter, as well as to

 preserve preserve the parts in contact, as far as their diseased state would admit of, that every advantage might be derived from the adhesive inflammation which I expected the remedy to excite. The above means were pursued night and morning. On removing the dressings upon the third day, the whole of the sphacelated parts was thrown off, which exposed a large portion of the tibia in two different places, to which pledgets of lint dipped in the fluid, were applied, which not only appeared to prevent the injuries usually attendant on those cases, (viz. discolouration and exfoliation) but on the contrary, in the course of eight days, the parts thus exposed were entirely covered by new granulations, and at the expiration of fourteen days, all the soft parts were perfectly reunited, and the surface of the ulcer reduced to a sore of about two inches and a half in diameter, with its granulations small, and compact, and of a beautiful florid colour. It is at present perfectly healed, and the patient, although a very lusty man, enabled, by the assistance of an elastic bandage, to attend to the duties of the ship.

"After the three first dressings, the Gastric Fluid of sheep only was used, not being able, from the ship being at sea, to obtain more from bullocks. The Fluid, if possible, should be procured fresh every day.

"To remedy his general ill health, an aperient medicine was first given; after which he took half a drachm of bark in two ounces of the decoction every six hours, with directions for him to make use of as many lemons and onions in the course of the day as his stomach would easily bear. It was astonishing to observe with what rapidity he regained his strength after the sphacelated parts were thrown off.

"I have since had the pleasure of finding the application of the Gastric Fluid succeed in more than a hundred cases of sphacelus, and the testimonies given of its peculiar good effects in these cases by Mr. Jones, surgeon of the Naval Hospital at Bastia, and by Messrs. Bead and Buck, two of his principal assistants, convince me of the importance of making its efficacy generally known.

"P. S. I have at present a patient in the Dolphin, a seaman belonging to the Barfleur, Vice-Admiral Waldegrave, who, from being confined to his bed by typhus fever, became excoriated in three different places, the whole of which terminated in extensive mortifications; the sloughs of which are entirely removed by the Gastric Fluid, and the patient sufficiently recovered to walk about.

---

*Observations on Mr. Simmons's Detection, &c. &c. with a Defence of the Cesarean Operation, derived from Authorities, &c &c. A Description of the Female Pelvis; an Examination of Dr. Osborn's Opinions relative to Embryulcia; and an Account of the Method of Delivery by Embryotomy.* Illustrated by numerous Engravings. By John Hull, M. D. Member of the Corporation of Surgeons, and of the Physical Society of London; of the Natural History Society of Edinburgh; and Secretary of the Literary and Philosophical

Philofophical Society of Manchefter. Pr. 9s. 6d. pp. 480. Bickerftaff and Clarke, London.

When men of fcience differ in opinion on fubjects of the utmoft importance to the medical profeffion, and to the world at large, and confine the debate clofely to the matter in queftion, the public are always benefited by it, unexpected lights fpring from the conteft, or at any rate the queftion has a better chance of being clearly underftood. But it becomes extremely painful to us, who are folicitous for the health of the public, and the promotion of fcience, to obferve two gentlemen of acknowledged ingenuity "*throwing dirt at each other*" with fo little good refulting from it; and we plainly perceive, if this quarrel *defcends* a very little lower, who *muft* decide "*when Doctors difagree.*"

Dr. Hull appears to have been at infinite pains in collecting materials for his book, and has furnifhed the reader with many particulars, which will gratify his curiofity; however, the Author's defign will be beft explained in his own words.

"From the fecond part both information and inftruction will, I hope, be derived. It contains the fubftance of a work announced for publication, under the title of a Treatife on the Cefarean Operation, previoufly to Mr. Simmons's unprovoked attack upon me. In this part I have criticifed a very curious letter, addreffed by Mr. S. to the Editors of the Medical and Phyfical Journal.—I have given a brief hiftorical account of the Section of the Symphyfis Pubis, and of the Cefarean Section, as practifed upon the dead and living female, with tables of all the fuccefsful and unfuccefsful foreign cafes, which I have been able to collect.—I have pointed out the circumftances, rendering the operation neceffary; and have laid down directions for accomplifhing the delivery under circumftances fuppofed to require the performance of the Cefarean operation; but in which it is neither neceffary nor admiffible. — I have entered into an enquiry concerning the moft proper time for having recourfe to the operation; the beft method of performing it; the fources of danger; and the fubfequent treatment of the patient.—I have endeavoured to make a fair eftimate of the comparative value of the life of the fœtus in utero, and the mother, according to the different conditions of the latter at the time of parturition. I have examined the opinions and precepts of Dr. Ofborn, relating to hyfterotomy and embryulcia; I have fhewn both by reafoning and actual experiments, that they are ill founded; and I have attempted to lay down rules and directions, that are lefs exceptionable.—I have entered into a difcuffion of the propriety and advantages of inducing premature labour, with the view of fuperceding the neceffity of embryulcia and hyfterotomy. —And I have introduced a fketch of the female pelvis in its natural ftate, with an account of the diftinctive characters of its deformities, and an enumeration of the caufes, by which thefe are induced, and of the means, by which their different degrees may be moft accurately determined."

The comparative eftimate of the value of the lives of the mother ther and fœtus, has been amply difcuffed by Dr. Ofborn, in his Effay on Laborious Parturition. In thefe Obfervations, Dr. Hull has examined the fubject with confiderable ability, and, we think, has juftly fet a higher value on the life of the infant than Dr. Ofborn, although he has not weakened (nor do we believe he means to leffen) our confideration for that of the mother. The learned author next proceeds to treat on the cafes that may require the Cefarean operation; and he fays, "It may be ftated in general terms, that every circumftance *which can render delivery per naturales vias impracticable*, ought to be confidered as requiring the Cefarean operation. What thefe circumftances are, writers are not agreed. For, whilft fome authors contend, that no cafe, except an extreme degree of deformity and contraction of the pelvis can render the operation neceffary, others enumerate a great variety of cafes, as indicating it.

"The cafes which I propofe to examine, are: 1. Deformity of the pelvis. 2. Wounds of the *uterus*. 3. Rupture of the *uterus* or *vagina*. 4. Extra-uterine conceptions. 5. Obliquity of the *uterus*. 6. *Hernia* of the *uterus*. 7. Tumours, &c. affecting the *os uteri* and *vagina*. 8. Strange or foreign bodies diminifhing the capacity of the pelvis. 9. Convulfions or Epilepfy. 10. Preternatural pofition of the *fœtus*. And 11. Monftrous conformation of the *fœtus*.—Of thefe I fhall confider the firft more particularly, and the others only in a general way"

On all thefe topics the author has taken a full view, and illuftrated his arguments with feveral plates, moft of which are well executed.

As much ftrefs in the whole of the debate has been laid on the cafe of Elizabeth Sherwood, related by Dr. Ofborn in his Effay on Laborous Parturition, we quote the following paffage, which ftates the *degree* of difficulty in that particular inftance in its proper light.

"Whether in any of the cafes, wherein the Cefarean fection has been had recourfe to in Great Britain, the delivery could have been accomplifhed by any means with more fafety to the mother, I am not able to determine; becaufe we are not in poffeffion of the dimenfions of the pelvis in all of them. But, in every inftance, where the dimenfions have been given, the degree of contraction was greater than in the pelvis of Elizabeth Sherwood, if this be determined by the fize of the largeft circle that can be formed in the fuperior aperture, which feems to me to be the moft proper mode of afcertaining their capacity. In the fuperior aperture of Sherwood's pelvis a circle may be formed equal to $1\frac{1}{4}$ inch; but we do not know that in any of the pelves, belonging to the poor women, who have undergone the Cefarean operation in Great Britain, a circle of the fame fize can be formed. We know that in the pelvis, of which I have juft given a defcription, the largeft circle, that can be formed in any part of the fuperior aperture, does not exceed one inch. It would therefore be by no means fair to infer, becaufe Dr. Ofborn afferts that he has delivered a woman by the crotchet, without deftroying her, whofe pelvis at the fuperior rior aperture, would admit of the formation of a circle with a diameter of 1¼ inches, that he can deliver a woman, with ſafety, by the ſame inſtrument, whoſe pelvis, in the wideſt part of the ſuperior aperture, will not permit the formation of a circle, the diameter of which is greater than one inch."

The author has collected with conſiderable induſtry a very full account of all the caſes of Ceſarean operation, and has reduced them into ſynoptical tables, with their fortunate or unſucceſsful terminations.

"The firſt of theſe tables comprehends a ſhort account of 110 caſes of the performance of the Ceſarean operation, and the third ſynoptical table, given in the 69th page of my Defence, &c. contains two, which, added together, make 112 ſucceſsful caſes. The ſecond table contains 27 unſucceſsful caſes, which, with the 15 caſes, contained in the firſt and ſecond ſynoptical tables, given in my Defence, &c. p. 66 and 67, and the caſe, which has ſince occurred in the Lying-in Hoſpital at Mancheſter, make 43 caſes, wherein the mother has died after the Ceſarean ſection. Now, if we ſubtract the number of the unſucceſsful from that of the ſucceſsful caſes, we ſhall find, that the latter exceed the former by 69."

For the Author's obſervations on the mode of performing the Ceſarean operation, we muſt defer our account till a future Number of our Journal.

---

*Some Obſervations on Vaccination, or the Inoculated Cow-Pox; by* Richard Dunning, Surgeon, Plymouth Dock, and Member of the Medical Society of that Town and Plymouth, pp. 122. London, Cadell and Davies.

The ingenious Author is a ſtrenuous advocate for the new inoculation, and appears to have taken conſiderable pains in inveſtigating its nature and mode of action; and juſtly obſerves, that "we may inoculate the ladies with the utmoſt ſafety in the advanced ſtages of pregnancy; and from another of its diſtinguiſhed traits—abſence of contagion—nurſes can be inoculated without danger of communicating diſeaſe to the infant, or vice verſa, the infant may be inoculated without injury, or ſubjecting the nurſe to any indiſpoſition or inconvenience whatever."

---

*Critical Survey of the lateſt Theories on difficult Dentition in Children.*

[ Continued from pp. 77—82 of our laſt. ]

We proceed now to give:

III. Dr. Wichmann's Theory, contained in his Ideas on Diagnoſtick, Vol. II. Hanover, 1797. pp. 1—81.

In paſſing over the medical writings and annals of all times, difficult dentition will be found to be almoſt unanimouſly acknowledged as a pathological phenomenon, and as the immediate cauſe of of many dangerous difeafes of children; and but few authors have hinted doubts againft an opinion fupported by the authority of its antiquity and by the force of prejudice. *Mercurialis*, who wrote about two hundred years ago, fays in his work, *De puerorum morbis Francofurt*, 1584, p. 312, mentioning dentition: "Sed ftatim nobis occurrit fcrupulus non fine animadverfione praelegendus, quomodo dentitio poffit effe morbus; compertum enim eft, naturam non intendere morbos neque favere, fed dentitio eft purum naturae opus, qua re non videtur morbus effe appellandus." However, he treats it as a difeafe, or, at leaft, as pains concomitant with that operation of nature. In modern times alfo, fome arguments have been fuggefted againft the general prevailing opinion, by Cadogan, (on Nurfing, p. 31,) by Armftrong (Difeafes of Children, p. 60,) to which his tranflator, Dr. Schaffer, has added his own obfervations, tending to prove the infufficiency of difficult dentition in producing fome difeafes of the infantile age, that are commonly deduced from that caufe. Notwithftanding thefe doubts, Cadogan, Armftrong, and Schaffer ftill confider it in too great a meafure as the fource of many infantile difeafes, not being able to prevail on themfelves totally to lay afide that opinion, although they have found it in fome refpect inconfiftent with their own obfervations and experience. Dr. Hecker, even though he denies thofe dangerous fymptoms to originate from the teeth cutting through, yet feems to regard dentition as a pathological phenomenon, afcribing it to an irritation in the mouth, which neceffarily muft be caufed by the teeth penetrating through.

During an extenfive practice of 30 years, Dr. Wichmann never could perfuade himfelf of the juftnefs of the idea on difficult dentition; and confirmed by his experience in the doubts he had always entertained, he attempted at firft to lay them before the public at large, however confcious of the hazardous tafk of encountering a matter that has hitherto ferved as an afylum ignorantiae to many phyficians, in explaining almoft every morbific fymptom by which infantile age is affected, and which fo often proves fatal to it. The frequent mortality of children, occafioned in the firft years of life by convulfions, is moftly attributed to the act of dentition, by which Hurlock (Practical Treatife upon Dentition, 1742) and other dentifts, account for them. Berdmore too (on the Diforders of Teeth and Gums, 1770, p. 192.) afferts, that more than half the children dying in the firft two years of their life fall a facrifice to dentition; and in this the greateft part of practitioners feem to have acquiefced, though none of them has ever given an exact account and explanation in what difficult dentition properly confifted, and the fuppofed morbid ftate hence arifing.

After thefe previous remarks, Dr. Wichmann proceeds to relate his opinion, which confifts in maintaining, that difficult dentition ought to be entirely exploded from the catalogue of difeafes, and that, as a pathological phenomenon, it exifts but in the fancy of phyficians. To fupport, and to prove this affertion, Dr. W. enters upon a difquifition and examination of the different fymptoms of difficult ficult dentition, and fhows how far they ftand the teft of criticifm.—The fymptoms fuppofed to attend difficult dentition may be divided into *topical* and *general*. Amongft the topical figns have been enumerated,

1. *A fwelling and hardnefs of the gums*, and this is looked upon as the fureft diagnofis of difficult dentition, particularly when children are not yet able to exprefs the fenfation of pain hence arifing. But it is obvious to remark, that fuch a fwelling will be always found in the healthieft children, who get their teeth in the eafieft way poffible, when a tooth is preparing to cut through; and, befides, if the fwellings were in the leaft of an inflammatory nature, it would certainly fpread itfelf over the neighbouring parts; but it is limited and fingle; elevations are only to be perceived, more or lefs, according to the number of teeth that are ready to break through. Inftances of inflammation and of tenfion of the gums, and of a fwelling of the cheek, which have been obferved by fome practitioners, at the time the dentes molares, or double teeth, are cutting, deferve hardly to be brought forward as an argument for difficult dentition, and muft rather be confidered as anomalous cafes, deviating from the natural courfe of nature, which differ from dentition, commonly fuppofed to be difficult, as panaritium and pernio, and are only defcribed as fomething extraordinary.

Moreover, this fuppofitious inflammation of the gums ought to be particularly evident and ftrong in the cutting of the eye-teeth, generally believed to be lefs eafy, and, therefore, principally dreaded, if it were a fignum pathognomonicum of difficult dentition; but on the contrary, at a clofe infpection, that fufpected red elevation will not even be found when thofe teeth are almoft hourly expected to get through at the end of the fecond year, and even when fome fymptoms of illnefs or difficult dentition appear at the time they are cutting through. Rednefs of the gums is likewife a precarious fymptom, as on the fpot where a tooth is appearing, the gums are moftly rather white than red.

2. *Pain of the gums* is counted amongft the moft important fymptoms of difficult dentition, and though children are not always able to exprefs the fenfation of it, and ftill lefs to point at the place whence it arifes, yet their exiftence has not been in the leaft doubted. However, children hardly complain of any pain on touching the gums; and even when they are of fuch an age as to be able to exprefs themfelves, and to denote exactly the very fpot where they feel the pain, particularly at the appearance of the eye-teeth, yet they never diftinctly complain of pains at thefe fufpected places, though they are moft clofely examined, and moft exprefsedly afked for them. Befides this, it ought to be prefumed that pains would be more perceptible in cafes where teeth break out in a preternatural way or direction; but this has not been found to take place when they grow one after another, forming a double row. Now, if in thefe cafes the jaw, the gums, the perioftium, the nerves, or to whatever elfe difficult dentition has been attributed, fuffer no affection at this time, at a ftate fo evidently violent and morbific, how can it be fuppofed to be fo, when the growth of the teeth is flowly proceeding in a natural way, and without any violence? We find, moreover, recorded by fome writers, that children come into the world with teeth that had cut through before their birth, where nothing may be thought prepared for fuch an action; and thefe children could have hardly lived if dentition was at all a violent and painful act.

Having pointed out how precarious and uncertain thofe fymptoms prove to be, it only remains to examine, whether the gums or the perioftium are really capable of giving pain; or whether, as it is commonly afferted, thofe parts are fufceptible of fuch a tenfion as not to emit the teeth without a painful fenfation. On this account practitioners never ought to be confulted, becaufe they admit and maintain the prefence of fuch a thing either without farther explanation, as Stoll fays, "Dens exitum quaerens gingivam tendit;" or they derive the pain a compreffione nervi, as Sauvage, well knowing that the gums fuffer incifion without caufing pain. Some afcribe it to a confenfus mufculorum, as Girtanner, (on Dieafes of Children, in German, p. 108.) to an irritation of the perioftium. Under thefe circumftances recourfe muft be had to anatomy for determining fatisfactorily the feat of pains, which are fuppofed to take place in dentition; however, we find almoft all anatomifts and phyfiologifts agree, that the upper part of the gums is nearly deprived of fenfation. J. Hunter, the firft author on this fubject, fpeaks, in his Hiftory of the Human Teeth, of the perforation of the gums, without in the leaft mentioning a perioftium; and, in explaining the pains attending dentition, he believes that there is an irritation which produces a diminution and abforption of the gums, at a time when, according to others, a fwelling of them is obferved. Blumenbach (Hiftory and Defcription of the Bones, in German) fays, that the teeth lie inclofed in a membrane, but he does not intimate that the upper part of them is alfo covered with it; and by the filence of other anatomifts, as Prochafka, (Annotationes Academicae) Albinus, Meyer, Loder, it may be juftly fuppofed that it does not exift after the birth. However, Haller expreffes himfelf very diftinctly, "Alveolus in foetu ampriffime patulus circa conformatum dentem connivet dum pars vitrea et abfque perioftio eminet," (Element. Phyfiologiae, T. iv. p. 23.) Soemmering (on the Structure of the Human Body, in German, 1791, T. i. p. 207.) fays, "While the roots of the teeth are growing longer, the teeth are protruded, and *bare of perioftium*, they penetrate the gums."—A ftill greater authority for this opinion is Alexander Monro, one of our firft writers on ofteology, who rejects the exiftence of a perioftium with plain words; "without the gums the teeth are covered with no membrane," (Anatomy of the Bones, Edinb. 1748, p. 149).

After giving thefe proofs of the non-exiftence of a perioftium, it is needlefs to dwell any longer upon this fubject; and it remains now to be confidered, whether the gums are thus pierced and irritated by the teeth as to produce thofe dangerous fymptoms of difficult dentition. It has already been intimated, that the beft and lateft phyfiologifts agree in denying almoft all fenfibility to the gingiva:

gingiva: but this is farther confirmed by the teftimony of furgeons, who think the incifion of the gums an operation attended with no pain, and confequently the gingiva is nothing but a fenfelefs fpungy fubftance, which eafily yields to the preffure and growth of the teeth. Hunter himfelf attributes but little fenfibility to the gums, remarking, that before the teeth appear they ferve in their place, which is likewife the cafe with old people. Another inconfiftency is, that it is propofed, by way of leffening the danger of dentition, to make the incifion, which is fuppofed to be attended with no pain, and yet a pain in the gums is taken to be the caufe of dangerous fymptoms! If then, thofe diagnoftic figns, fo generally confidered ftrong and inconteftable, are proved to be uncertain and illufive, and if at the fame time no pain can arife from the gums, the fuppofition of which has been employed as a caufe of all the different fymptoms of difficult dentition, it muft be candidly allowed that this pathological explanation is deficient and uncertain.

3. *Salivation* is another fymptom attending dentition, on which phyficians rely fo much, that at perceiving this in a child they do not require any other fymptom, and hardly think it worth while to examine the mouth; but if they did, they would very frequently find falivation was occafioned by aphthae or the thrufh, of which it is, as well as pains, a common fymptom; and though they are not always met with, yet they are more frequently the caufe of this falivation than has hitherto been thought; and in cafe they fhould not be there, it is more natural to derive the falivation from a flight irritation of the falival glands than from dentition.

There is ftill another fymptom likewife apt to deceive, and that is, *when children often move their hands towards the face.* Of many inftances, the following may prove the truth of its being fallacious. A child, two years old, had been fickly about a fortnight from an unknown caufe; it loft appetite, fleep, and became feverifh; it fhrieked fometimes, and often brought its hands towards the face, when Dr. W. was fent for. On undreffing and examining the child, to difcover the caufe, an excoriation in the ear was found, which being removed the child got quite well again. In difeafes of children, therefore, nothing is more neceffary than exact examination of the whole body, which will frequently, if not always, fhow how fmall a fhare dentition has in producing them, and how eafily one may be deceived in thinking that the pain arifes from the place to which children appear to point. Of the numerous inftances by which this may be demonftrated, a very interefting obfervation of Dr. Lodemann, of Hanover, deferves to be mentioned. A boy, five months of age, became fickly, cried often, without having any fever, and the caufe was affigned to teething. Soon after convulfions, trifmus came on; he loft the power of fwallowing; nothing but external remedies could be applied, and he died after forty-eight hours. In diffecting the body, the true caufe was difcovered to be a preffure of the left tefticle in the abdominal annulus.

Aa 2 After

After having ftated what can be faid of the topical figns of difficult dentition, Dr. W. proceeds to confider the general fymptoms and difeafes afcribed to it. When all thefe difeafes are impartially examined, they will be found not to differ in the leaft from other affections, daily obfervable in the firft four months of life, where teeth are not yet fufpected, and after the fecond year when moft teeth have appeared. They feem, therefore, to admit a more natural explanation: By confidering them as idiopathick (aphthae) and diarrhoeas, colics and vomition are much eafier explained from a corrupted milk, and an inflammation of the eyes from a fcrophulous difpofition. It feems, likewife, inexplicable how a kind of dyfentery can originate from dentition, as is afferted by medical authors, particularly when no pain in the mouth, and other topical fymptoms are perceived, and when fimilar evacuations occur in children of five or fix years of age It is more probable that it arifes from a topical caufe, or any other lefs remote from the inteftines. A ftrangury and retention of urine are alfo fuppofed to be confequences of dentition in female children; but they really are only owing to an excoriation and exulceration of the genitals, and eafily yield to a topical treatment. A lethargic ftate is more probably caufed by a hydrocephalus internus than by dentition. The gonorrhoea, which is fometimes obferved at the time of dentition, is nothing elfe but an accumulation of matter, fecerned by the glandulae tyfoni, and is a topical affection foon removed by injection under the prepuce. With refpect to the fever incident at the period of teething, it may be obferved, that many circumftances can occafion it, as children, even in that age, are fubject to every febrile affection; and Rufh has feen the yellow fever in a child of four months, without any neceffity of affigning dentition as the caufe of it; and it is fcarcely pardonable to neglect other remedies with the idea of its being merely the confequence of the irritation of the teeth; and befides, it does not appear that it is removed when the teeth have cut through; and frequently, when the fever has been attributed to dentition, no teeth have appeared. There are alfo fome febrile affections remarked as fymptoms of a latent fcrophulous difpofition, and thefe are frequently miftaken for the teething fever.

Of all morbid affections by which children are afflicted, during the time they are cutting their teeth, none have been fo generally, and without exception, attributed to dentition as convulfions; but when we confider that moft probably they are owing to caufes more frequently occurring, to a momentary corruption of the milk from paffions of the mind, to other difeafes of the nurfe, to the reftoration of fluxus menfium, &c.; and when we farther confider that all important difeafes generally begin with convulfions in the infantile age, it may be concluded how precarious the idea will prove in the practice. The following cafe may, moreover, prove the fallacy of this fymptom:—A healthy boy, feven months old, was feized with convulfions and pains, which recurred in a fhorter or longer fpace of time. Gentle purgatives, clyfters, and blifters behind the ear, removed removed theſe affections, but after four weeks no elevation or penetration of a tooth was to be perceived.

Such are the ſymptoms, the pathology, and diagnoſis of difficult dentition, of which it has been attempted here to point out the deficiency and fallacy. In ſurveying the method of cure delivered by medical writers, we meet with the ſame inconſiſtency, and the groſſeſt empirical uncertainty. Hurlock has collected all ſuperſtitious remedies that were in uſe in his time, and has beſides enriched it with the hare's-brain, which was employed to ſoften the gums or to promote the growth of the teeth. Hard and ſoft things were indiſcriminately given to children to bite; and, in ſhort, remedies were employed which only ſuperſtition could have invented, and ignorance and indolence kept up. But of all remedies, none has been ſo generally received and ſupported by the authority of practitioners as the inciſion of the gums, of which Ambroſius Paré, an eminent French ſurgeon, of the ſixteenth century, ſeems to have been the inventor. The uſe of this operation was afterwards much confirmed by Hurlock, though rather too far extended by him, as he applied it at the age of three years; and once even when the child was but ten days old. Berdmore, a known dentiſt of his time, and other eminent practitioners, have adopted it. Underwood (Diſeaſes of Children, 1784, p. 95) recommends it very much as being attended with no pain, though pain and inflammation are ſaid to take place at this very ſpot; and, beſides this, he derives it from the perioſtium or membrana nervea, covering the teeth immediately under the gums; but it has been ſufficiently ſhown that no ſuch thing exiſts.

[ To be concluded in our next. ]

---

*Elemens de Myologie et de Syndeſmologie, par Thomas Lauth,* 1 *vol. à Bâle, chés Decker, à Paris; chés Koenig, et à Straſbourg. VI. année de la Republique.* 1798. pp. 204.

Amongst the number of anatomical compendiums that have been publiſhed in different countries, the preſent deſerves to be recommended on account of its accuracy, conciſeneſs, and the order which the author follows, as well on account of containing a complete literature, and references to the beſt figures. The whole is divided into ſeveral lectures, of which this volume contains but eight, beſides an introduction, in which ſome general remarks on the ſtructure, action, diſtribution, and denomination of the muſcles, on the folliculi mucoſi, and the ligaments, on the literature of this branch of anatomy, are previouſly explained. The redneſs of muſcles, which the author conſiders as eſſential to them, merely depends upon the red part of the blood contained in their veſſels, and eaſily diſappears by putting them in water for ſome time, and beſides the muſcles of animals, without red blood, (animalia ex ſanguia,) as inſects, are white; that therefore the red colour cannot be conſidered as an eſſential property of muſcular ſtructure. The word *muſculus* he thinks rather to be derived from μυειν (contrahere) than than from μυς, as it is generally ſuppoſed. In deſcribing the muſcles, he proceeds almoſt according to *Albinus's* method, though it ſeems a little inconvenient, that the deſcription of thoſe muſcles which extend to different parts, is ſeparated in ſuch a way, that, for inſtance, the greateſt part of the latiſſimus dorsi is deſcribed with the muſcles of the back, but its ſmaller portion, and tendon with thoſe of the arm. It is, however, very uſeful to the beginner, that the author has prefixed to the deſcription, the beſt mode of preparing each part anatomically in the following order:—*preparation, ſynonimous terms, ſtructure and inſertion, and uſe.* In mentioning *Veſalius's* work, he ſupports an opinion of *Albinus,* that the plates to it were not done by *Titian,* but by *Jean Etrenne,* becauſe he thinks it not likely that *Titian* ſhould have attended to anatomy at the advanced age of 62, when young *Veſalius* was publiſhing his work; being, beſides, very much occupied with pictures, that were ordered to be made by him; and it is moreover obſerved, that great painters are ſeldom able to draw objects of natural hiſtory and anatomy with accuracy, which can only be acquired by continual practice.

---

*Chemiſhes Handwoerterbuch, &c.* i. e. *Manual Lexicon of Chemiſtry, compoſed after the lateſt Diſcoveries, by* Dr. Dav. Lewis Bourguet, Profeſſor of Chemiſtry, at Berlin. *With a Preface of* Dr. Sigm. Frederick Hermbstaedt. Vol. 1. A—E. 1798. Vol. 2. F—K. 1799. Berlin for Bemigke. Pr. 4 rixdollars, or about 16s.

Although we poſſeſs in the Chemical Dictionary of Macquer, a work which is full of the beſt chemical information, and for the time in which it was written, extremely well performed, and calculated to give a complete and general ſurvey of chemiſtry, yet the aſtoniſhing progreſs which that ſcience and art has made of late, and which is almoſt daily increaſing, ſeems to have rendered it neceſſary to compoſe a new work of the kind, whoſe chief object of utility and accuracy, would of courſe conſiſt in having collected all diſcoveries and facts in chemiſtry that are known to the preſent times. Mr. *Bourguet* has therefore a claim to the praiſe of the public for having undertaken this taſk, of which, as far as we are able to judge, he has hitherto ſufficiently acquitted himſelf. However, it would not have been amiſs, if in ſome reſpects he had attempted to be more conciſe, which we think is a neceſſary requiſite for a work, whoſe object is to convey information in an eaſy way. More care ought alſo to have been taken in the correction of the preſs.

*Deſcription*

Mr. Humbolt's
Anthrasometer

See Page 179.

Published, Oct. 1.1800, by R Phillips, 71, St. Paul's Church Yard.

*Description of Mr.* HUMBOLDT's *Anthracometer, or an Inftrument for meafuring the Quantity of Carbonic Acid contained in the Air.*

[ With an Engraving. ]

THIS inftrument confifts of a very ftrong glafs tube, from 3 to 4 lines wide, and about 12 inches long; and ends in a globe of 1.2 or 1.3 inch in diameter. The lower three inches of the tube are bent in fuch a way, that the diftance of the globe from the cylinder is no more than 6.3 inches, for the fake of putting it into a narrow glafs of water. The tube *a b* muft be of equal width, but it is indifferent whether it be wider in *c* or *d*; however, it is better when the capacity of the whole cylinder does not exceed 2 or 2.5 cubic inches. In *e* it is divided in two fections, of which the upper one muft remain 7 inches long; both parts are joined by a metallic cylinder, fo that they may be unfcrewed; but no water muft get through. The upper end of the glafs tube is cemented into a metallic cylinder, 6 inches, high *f*, which has, on its outfide, nine fmall worms of a fcrew. Its mouth is conically turned out, and into this a mufcle-fhell-like valve is put, of the thicknefs of one or two lines. Another metallic cylinder, *h*, 5 lines high, fhut by a plate above, *k l*, and excavated as the box of a fcrew, exactly fits the tube as a cover. To increafe the preffure, the plate *k l* has a hole in the middle, and another fcrew preffes the valve to the mouth of the tube.

The ufe of this inftrument is very fimple. Fill it with *liquid cauftic ammonia*; pour out of the tube, *a e*, as much as you are going to examine of the air, and mark with a pair of compaffes the length of the column of air on a fcale; then fhut the valve, and let the air go into the globe; and the carbonic acid contained therein, will, on account of the greater furface it finds, combine itfelf entirely with the cauftic ammonia. The latter will begin to fink in the tube; and when this procefs is over, open the valve and fill the whole tube with it. Let the air from the globe pafs into the tube again, which is then unfcrewed at *e*, under water; and the upper piece muft likewife be immerfed till the water is equally high at the infide and the outfide. The remaining part of the air, fubftracted from the above quantity, fhews the proportion of carbonic acid. Inftead of the ammonia, kali may, perhaps, be preferable, as it is more eafily prepared. The inftrument may likewife be ufed for meafuring oxygen gas.

---

*Remarks on the Application of cold Water in Amaurofis and Weaknefs of Sight; by Dr.* BEER, *Occulift at Vienna. (Extracted from* Arnemann's Magazine for Surgery, *Vol. II. No. I.)*

Cold water is undoubtedly one of the moft powerful and fureft remedies againft the weaknefs and inactivity of the veffels and optic nerves, which generally remain after a ftrong congeftion of the blood towards the head and eyes. It is therefore employed with the greateft fuccefs in that fpecies of amaurofis, caufed by a congeftion gestion of blood to the head, after sufficient quantities of blood have been taken away by a general and topical venæsection, to remove the subsequent distention and weakness of the vessels. How useful the external application of cold water often proves to be in amauroses, originating from a general debility of the body, or a topical weakness of the eyes, is known to every practitioner.

Persons who have suffered vehement inflammations of the eyes frequently retain a dim and weak sight, though no visible fault can be discovered in the eyes themselves. They sometimes are entirely cured in a few days, by the external use of cold water. It likewise happens after the extraction of the cataract, that the operated persons complain of black serpentine lines, marks, and dust, floating before the eyes, and hindering the sight; which symptoms always yield to the use of cold water.

The application, however, of this excellent and simple remedy requires some caution. As long as any symptom of congestion is perceived, it should be carefully avoided. The patients, indeed, will feel a momentary relief and amendment in the sight soon after the application, but a great tension in the eye-ball, a decrease or entire loss of sight, and sometimes vehement ophthalmies follow the inconsiderate use of cold water. When, on the contrary, the face of the patient is pale, and the white of the eye not red or yellowish red, but when only a few turgescent blood vessels are remarked on it, when motion causes no head-ach, nor increases the dimness, when sparks or other shining corpuscles appear no more before the eyes, and the patient perceives no tension, or any other particular sensation in moving the eyes, cold water may be used with safety and advantage.

In applying it, a gradual proceeding is to be observed. At first, head and eyes should be covered with a cloth dipped in cool water, which must be frequently repeated. Some days after they may be bathed with colder water. After having performed this for eight or ten days, it may be applied in a more powerful way by means of a syringe, with which the water is brought to the eye-lids, eye-brows, temples, and, if the patient can bear it, to the eyes themselves.

The usual mode of applying the water in these cases by means of small cups of glass, or earthern ware, can never have any effect, by the way of coldness, because the water gets too soon, in this way, a considerable degree of warmth; but the other application has the double effect of bringing the water sufficiently cold to the part, and by causing an efficacious commotion in the eye itself.

To apply the water in this manner with greater ease and more advantage than by a syringe, a very convenient instrument has been invented by Dr. *Beer*, which is very well adapted to the purpose. (see the plate.) The whole is made of copper or tin, and consists of a box, in which is inclosed a smaller vessel, made of pewter, *F*, which contains about five or six pounds of water. The space *G*, between the two vessels, is filled with broken pieces of ice, and the degree of cold may be increased according to circumstances, by by adding ſalt or ſal ammoniacum to it. The tube is divided into four parts, which are well joined by ſcrews, *D*, by which means they can be conveniently put into the box, when the inſtrument is not wanted. By turning the cock, *E*, the water may be ſtopped, or by opening it more or leſs, the force of the fall of the water be increaſed or diminiſhed, as it ſeems proper. (For this purpoſe, it would be very convenient, if the ſpout of the tube *I*, could be unſcrewed, ſo that different ſpouts with larger or ſmaller openings might be put on, as the former would tend to leſſen the force of the water, and the latter to increaſe it. Another ſpout, made like the roſe of a watering-pot, and perforated with many holes, would be well calculated to give a greater ſurface for the water to act upon.)

The whole inſtrument is about five feet long, and may be ſuſpended in ſome convenient place of any apartment; but it ſhould be high enough that the patient need not, in uſing it, bend the head too much down. The lid, *C*, is faſtened on the veſſel in *B A*, as appears from the plate.

In obſtinate caſes of Amauroſis, where cold water is preſcribed, it may be uſed as a dropping bath, by ſcrewing the ſpout, *H*, to the tube, inſtead of the curved end. The cock may be turned in ſuch a way as only to emit the water by drops, which fall either on the eye-brows or temples; and the effect of this powerful remedy can be leſſened or ſtrengthened, according to the height from which it is applied. The commotion and pain, cauſed by this mode of application, is generally ſo vehement, that many patients will not bear it. However, its efficacy is very great, and caſes, where it proves without avail, muſt be conſidered as doubtful and moſtly incurable, and very rarely yield to other exciting remedies, as electricity, &c.

The uſe of this convenient inſtrument might be made more general and common in bathing and ſtrengthening the eyes, and would do a great deal of ſervice, particularly to thoſe whoſe ſight is weakened by reading, lucubration, or by any other means. To theſe it would prove a very uſeful and commendable piece of furniture, and for that purpoſe the above mentioned alterations in the ſpout would, perhaps, be requiſite; the ſmaller veſſel, *F*, might then be taken out, as the degree of cold need not be ſo great for common purpoſes.

## Figures and Tables

**Fig. 2. Fig. 3. f1:**